# Comparing three methodologies for network analysis of human [^11^C]glyburide whole-body PET data: *d-*networks, *s-*networks, and *ΔPCC* networks

**DOI:** 10.1186/s13550-025-01348-x

**Published:** 2025-12-31

**Authors:** Abigail F. Hellman, Paul S. Clegg, Solène Marie, Nicolas Tournier, Adriana A. S. Tavares

**Affiliations:** 1 School of Physics and Astronomy, University of Edinburgh, Edinburgh, UK; 2 BioMaps, Service Hospitalier Frédéric Joliot, Université Paris-Saclay, CEA, Inserm, CNRS, Orsay, France; 3 University/British Heart Foundation (BHF) Centre for Cardiovascular Science, The Queen’s Medical Research Institute, University of Edinburgh, Edinburgh, UK; 4 Edinburgh Imaging, University of Edinburgh, Edinburgh, UK

**Keywords:** Whole-body PET, Network analysis, [^11^C]glyburide, Dynamic PET

## Abstract

**Background:**

Dynamic whole-body PET and total-body PET both supply large datasets that include multiple organs, opening the opportunity to study systems biology via appropriate analysis. Network analysis, commonly used with brain imaging, is applied here with whole-body PET to compare data from different tissues and subjects before and after precipitating a pharmacokinetic drug-drug interaction. This is done with [^11^C]glyburide PET, a radiotracer whose tissue distribution is mediated by organic anion-transporting polypeptides (OATP) transporter function. OATPs control the uptake of drugs, primarily into the liver. We examine three methods of network analysis to evaluate the effort, efficacy, and potential applications for further research use. This was performed with 22 dynamic [^11^C]glyburide whole-body PET scans of healthy humans. This includes 13 baseline scans, and 9 after infusion of rifampicin, a potent OATP inhibitor. All methods use Pearson correlation coefficient. The first method generates “*d-*networks” using dynamic data from one region. The second method generates “*s-*networks” using static data from multiple regions, but not all regions. The final method generates “Δ*PCC* networks” with static data from all regions. The first two methods compare subjects, whereas the third compares regions.

**Results:**

The *d-*network differentiates control and rifampicin subjects within the primary region of interest, the liver, but the differentiation is not complete, and there is still some correlation across subjects (*r>0.65, p<0.05)*. The *s-*network completely distinguishes trial groups but requires removing anomalous data from consideration (*r>0.65, p<0.05)*. Because *d-* and *s-*networks require selecting which regions are included, they are informed methods. In contrast, the Δ*PCC* networks are uninformed. This method utilises all data and still highlights significant regional variations between the two groups, such as in the liver, while remaining robust to confounding variation (|*ΔPCC*|*>0.18, p<0.05)*.

**Conclusions:**

All three methods of network analysis with whole-body PET were successful at identifying key information in the dataset. Each method required different levels of processing and interpretation, making them applicable in different scenarios depending on whether the PET data is dynamic or static, if the kinetics in regions of interest are well understood, and whether the study is focused on comparing between treatment groups or regions.

**Supplementary Information:**

The online version contains supplementary material available at 10.1186/s13550-025-01348-x.

## Background

While conventional positron emission tomography (PET) scanners only image one area of the body at a time, they can be used to capture whole-body images through a method known as whole-body PET (WBPET), where the subject is moved through multiple bed positions in the scanner. WBPET can also be performed dynamically (WB-4D), by performing the multi-bed acquisition repeatedly [1]. WB-4D PET acquisitions provide ample kinetic data including multiple organs, making this a valuable tool for studying systems biology at the physiological level [2]. This paved the way for the development of total-body PET (TBPET) scanners, with a large field-of-view to enable WB-4D PET acquisition with improved time-framing.

WB-4D PET enables exploring the molecular mechanisms underlying systemic drug delivery [3, 4]. Research suggests that membrane transporters at the blood-tissue interface control the delivery of drugs to tissue [5–8]. WBPET using radiolabelled analogues of drugs can provide insight into transporter expression (9). For example, transporters of the Organic Anion-Transporting Polypeptide (OATP) family are known to mediate the uptake of many drugs into the liver [10]. While many OATPs are thought to be liver-specific, some extrahepatic expression has been observed [11, 12]. While the main expression is observed in the liver, extrahepatic expression of OATPs makes these transporters an interesting subject for WBPET imaging. [^11^C]glyburide is a recently developed PET probe for whole-body OATP function and drug delivery, as glyburide is a substrate for several OATPs [9, 13–18]. WB-4D PET studies, however, have shown no evidence of extrahepatic OATP-mediated transport of [^11^C]glyburide [9, 18]. Nonetheless, it is a metabolically stable radiotracer with high sensitivity to OATP inhibition. This was tested using the safe, potent OATP inhibitor rifampicin [7, 13, 14].

Network analysis, a correlation-based image analysis method, has been applied extensively in brain studies using magnetic resonance imaging (MRI), single-photon emission computed tomography (SPECT), and PET [19–21]. It generates representative networks, also called graphs, displaying quantified comparisons between data from different tissues and subjects. These networks have elucidated the complex structure and functionality of the brain, revealing both spatial connectivity and signal correlations between regions [22–24]. Network analysis has served as a popular, robust mathematical method for studying brain connectivity, providing insight into normal cognition of healthy individuals and serves as a framework for understanding disease mechanisms [25, 26]. For example, brain diseases including schizophrenia and Alzheimer’s are associated with distinct structural and functional network alterations [19]. Furthermore, networks have detected subtle disruptions in diseased brains, such as those with mild cognitive impairment, that could not be detected with other analysis methods [27]. As brain connectome research advances, network analysis holds promise as both a diagnostic and prognostic tool.

Similar work is being done to apply network analysis to the whole-body to understand inter-organ interactions in healthy and diseased states [28, 29]. Group-level network analyses of static TBPET have shown that lung cancer patient networks differ from healthy subjects, with decreased efficiency that suggests disrupted coordination between organs [30, 31]. Furthermore, bone-specific WBPET networks were shown to predict lung cancer patient survival where conventional standardised uptake value (SUV) peak analysis could not [32]. Systemic network analysis can also inform potential new research targets, such as static TBPET networks implicating the role of the brain-white adipose tissue axis in diabetes mellitus [33]. Similarly, dynamic murine TBPET networks show that mice lacking phosphatase, orphan 1 – a bone mineralisation enzyme – exhibit different metabolic networks from their wild type counterparts [34]. A range of other WBPET studies, including static and dynamic data, have used network analysis to characterise healthy and disease states, advancing systems biology [29, 35–37].

Across the noteworthy amount of research involving network analysis with WBPET, many different methods have been implemented. Some compare within subjects while others look across subjects, and many use static data while some incorporate dynamic data. While network analysis with both static and dynamic TBPET data has been shown to outperform conventional standard uptake value (SUV) analysis, methodological differences have not been so thoroughly explored [32, 34, 36]. Extensive efforts have been made to describe and standardize nomenclature and methodology in brain molecular connectivity studies, but require further work at the whole-body level [21, 25, 26, 38, 39]. Here, we seek to clarify and standardize some key methods, exhibiting their use cases and outputs.

We propose, demonstrate, and compare three network analysis methods with [^11^C]glyburide WBPET in healthy subjects, some rescanned after rifampicin infusion. This novel work seeks to show how networks, traditionally used in neuroimaging studies, can be applied to PET to study physiology at a systemic level. Furthermore, we explore three different methods to example how each one works with different data types and for different research uses. The first method utilises dynamic data to produce single-tissue networks called “*d-*networks”. In practice, these networks would be useful for studies with dynamic data to perform group-level analyses or assess tracer kinetics. The second method employs static data from the entire whole-body scan to compare subjects at a systems-level. The results will be called “*s-*networks”. These networks would be useful for analysing large datasets at the group-level, potentially uncovering group characteristics. The third method also uses static data and compares both at the group- and organ-level simultaneously, and the output will be “*ΔPCC* networks”. *ΔPCC* networks can be used for individual-level analysis, with the prospective benefit of uncovering what makes a subject (or subjects) different from a known group. The *d-* and *s-*networks are “informed” methods, as they require selecting the data included in the networks based on known patterns in the dataset – i.e., performing the *d-*network method, which produces single-tissue networks, only with liver data as [^11^C]glyburide is predominantly expressed in this tissue. Each method suits different research purposes depending on the data type, whether an informed or uninformed approach is necessary, and the comparison scale (region-, subject-, or group-level).

## Materials and methods

### Subjects and study design

The imaging followed the protocol described in Marie et al. [18], approved by an Ethic Committee (CPP IDF5: 17041, Study registration EudraCT 2017-001703-69) and conducted in accordance with the 1975 Declaration of Helsinki. All subjects signed an informed consent form. Sixteen volunteers underwent baseline imaging, and ten underwent a subsequent rifampicin scan. Three baseline and one rifampicin scan were excluded due to scan length and region discrepancies. The control group included *n=*8 males (40±18 years) and *n=*5 females (60±2 years). The rifampicin group included *n=*6 males (31±16 years) and *n=*3 females (60±1 years).

### Image acquisition and processing

Whole-body dynamic PET (WB-4D PET) acquisitions with intravenous [^11^C]glyburide were performed using a Signa® PET/MR scanner, according to the protocol described in Marie et al. (GE Healthcare, Waukesha, WI, USA) [9, 18]. All subjects received the first injection of [^11^C]glyburide (167 ± 56 MBq i.v.). For subjects who underwent a subsequent scan (174 ± 53 MBq i.v.), this was performed 3 h after the first acquisition and the rifampicin infusion was within 45 min of [^11^C]glyburide injection (9 mg/kg diluted in glucose 5% perfused, within 45 min of [^11^C]glyburide injection, Rifadin®, Sanofi-Aventis, Gentilly, France).

The WB-4D PET acquisition began with a 3-minute dynamic mono-bed acquisition positioned on the abdomen (16 frames of 10 s). Then, multi-bed whole-body acquisitions were performed repeatedly over at least the next 30 minutes (5 bed positions, at least 11 frames). The images were reconstructed using an iterative 3D reconstruction algorithm. Data were corrected for decay, attenuation, and scatter. Attenuation correction was performed using the MR data. The PMOD software was used to delineate volumes of interest (VOIs) using PET and/or co-registered MR for nine tissues: left and right kidneys, liver, aorta, aorta wall, ventricle, myocardium, gallbladder, and pancreas (version 3.9, PMOD Technologies LLC, Zurich, Switzerland).

The spleen was not included in analysis due to one subject having had a splenectomy; however, the incomplete dataset is included in Supplementary Fig. 1. The testis was also omitted due to the inclusion of female subjects. Other regions such as the brain, bladder, muscle, and eyes were not included as only late-time information existed for these regions. To directly compare network analysis methods using both dynamic and static data, we retain only those regions with activity data throughout the full scan time.

Data were extract as time activity curves (TACs), expressed in SUV corrected for injected dose and body weight. As many of the images had different scan and frame lengths, data were linearly interpolated for standardization. Although interpolation cannot be performed without assumptions about the data – primarily that it is monotonic – linear interpolation was chosen because it does not smooth the data in the same way as other interpolation methods. All scans were interpolated from 14 to 2416 seconds with 240 equally spaced points. This standardized all data to about 40 minutes in length, sampled every 10 seconds. Static data is the average uptake over the last ten minutes of interpolated data.

### Network analysis

Each network analysis method was performed using the Pearson correlation coefficient (*PCC*), which measures linear correlation. This is done in Graphia (version 5.0, https://graphia.app/) for the first two methods, and Python for the third [40]. *PCC* requires the same number of data points in each dataset being compared, making the earlier linear interpolation step necessary; however, *PCC* is a normalised measure giving the average linear relationship between data, so linear interpolation is not expected to introduce significant bias.

### Region-specific intersubject dynamic network (*d-*network) analysis

This first method of network analysis involves calculating the *PCC* between subjects’ TACs within a given region. Given this method uses dynamic data, we refer to these as *d-*networks. As we are calculating the correlations between subjects, this is an intersubject method. To generate a *d-*network with Graphia, the PET data must be in tabular format in a comma separated values (CSV) file, with rows representing subjects and columns representing time points. Graphia then calculates the *PCC*, *r*, between each TAC and displays a visualised graph. In the *d-*network graph, nodes represent each subject TAC for the given region, and edges between nodes represent significant correlations (*r>0.65, p<0.001*) [41, 42]. The edges are weighted to the *PCC*. A k-nearest neighbours edge reduction algorithm was also applied (*k=3*). This reduces the total number of edges by retaining only the *k* strongest weighted edges per node and pruning the rest, unless there are more than *k* of equally strong weight [40, 43]. An example of the CSV file setup and a diagram of the method is given in Supplementary Fig. S1. One *d-*network was generated per region: liver, aorta and ventricle, hepatic vein, kidneys, aorta wall, myocardium, gallbladder, and pancreas.

### Whole-body intersubject static network (*s-*network) analysis

This method calculates the *PCC* between subjects using static data, so we refer to these as *s-*networks. Each subject is represented by a set of discrete SUV values for each region analysed. To generate these *s-*networks in Graphia, the tabular CSV files are formatted to have rows representing subjects and columns representing regions. This method was first performed including all nine regions, then again with the gallbladder data removed from consideration. The reasoning for this is expanded on in the Discussion section. A *PCC* threshold and edge reduction algorithm were applied (*r>0.65, p<0.05, k=3*). An example of the CSV file setup and a diagram of the method is given in Supplementary Fig. S2.

### Whole-body intrasubject static Δ*PCC* networks

**Fig. 1:**
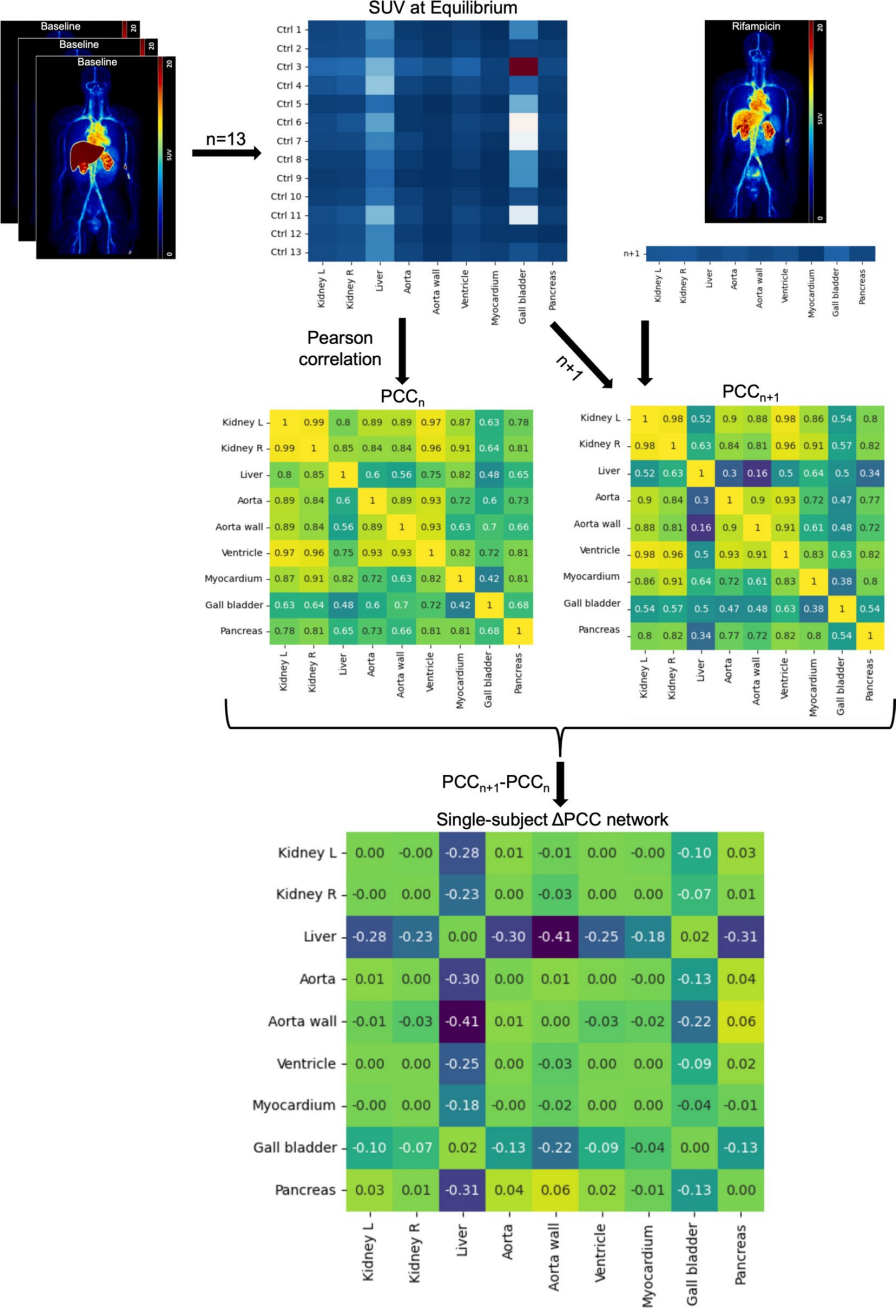
Method for deriving intrasubject Δ*PCC* networks. Starting with the equilibrium SUV from the PET scans of *n=13* control subjects (also in Fig. 2b), the PCC is calculated from the subject curves between each of the 9 regions (*PCC*_n_). Data from one of the rifampicin subjects is then added in and the calculation is redone, now with *n+1* subjects (*PCC*_n+1_). The difference between the two networks is then found (Δ*PCC* network for one rifampicin subject)

The Δ*PCC* networks also use static data, but the *PCC* is no longer calculated between subjects. This is now intrasubject, as calculations are performed at the group-level. Following along with Figure 1, we first set up the data in a matrix format like the *s-*network method. Rows represent different subjects and columns represent different regions. This only includes the *n* control subjects to start, though, unlike the *s-*network method. The *PCC* between every region is then calculated and the results are saved as a new matrix entitled *PCC*_n_, the reference network. Next, one rifampicin subject is added to the original matrix, giving a matrix with *n+1* rows. The *PCC* between every region is again calculated, resulting in a matrix *PCC*_n+1_, the perturbation network. Finally, the difference of the two matrices is calculated, resulting in our Δ*PCC* network (*|ΔPCC|>0.17, p<0.05*). This process is repeated for each rifampicin subject individually. To get an average Δ*PCC* network for all subjects, a significance threshold was first applied to each individual Δ*PCC* network before calculating the average of each matrix element.

## Results

### Liver-isolated intersubject *d*-networks differentiate control and rifampicin groups

**Fig. 2:**
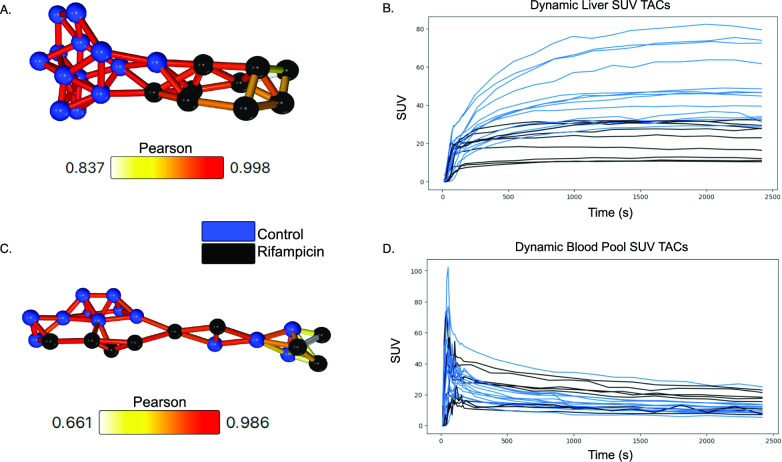
Intersubject *d-*networks with Pearson correlation **a** *D-*network where each node represents the liver SUV TAC from a single subject **b** Liver SUV TACs for all subjects **c** *D*-network where each node represents the blood pool (aorta + ventricle) SUV TAC from a single subject **d** The blood pool (aorta + ventricle) SUV TACs for all subjects (n=13 control, n=9 rifampicin)

When the interpolated SUV TACs were compared between subjects one region at a time, the liver-specific *d-*network showed distinct separation between the control and the rifampicin-treated subjects (Fig. 2a). The network is comprised of one component containing correlations between the two groups, but each are on separate sides of the component. The control group also has denser connections that are typically of higher significance compared to the rifampicin group; however, all correlations are highly significant in this network (*r>0.65, p<0.001*). Meanwhile, a *d*-network of the blood pool data (a combination of aorta and ventricle data) shows no separation based on trial group (Fig. 2c). This is the same for other regions as well (Supplementary Fig. S3).

### Informed intersubject *s-*networks separate control and rifampicin groups into separate components

**Fig. 3:**
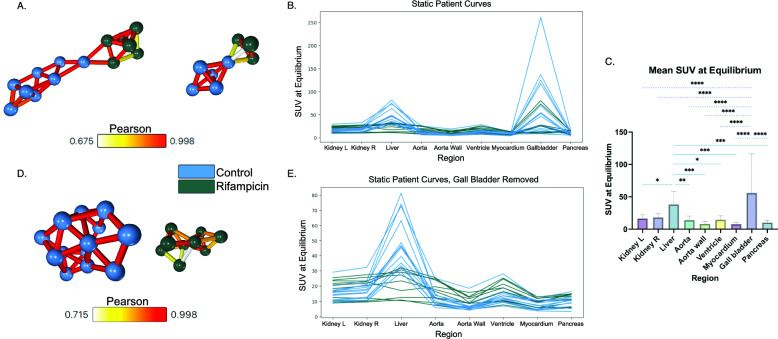
Intersubject *s-*networks with Pearson correlation **a***S*-network where each node represents a discrete set of SUVs from each region for a given subject, with all regions considered **b** The curves used to generate the network in** a c** Comparison of the mean equilibrium SUV across each region with one-way ANOVA returned significant differences between the liver/gallbladder and every other region. Data are presented as mean±SD (*n=22*) **d***S*-network where each node represents a discrete set of SUVs from each region for a given subject, without the gallbladder data. **e** The curves used to generate the network in **d** (*n=13* control, *n=9* rifampicin)

Intersubject *s-*networks containing data from every region fail to differentiate control and rifampicin groups (Fig. 3a). The liver and gallbladder show the greatest uptake variation (Fig. 3b). The mean SUV of both regions are significantly different from most other regions when all subjects are considered (Fig. 3c, r*>0.65, p<0.05*). However, other regions do not significantly differ from each other. High uptake in the liver is expected with [^11^C]glyburide, with reduction expected after rifampicin infusion. The gallbladder, however, is known to have extreme person-to-person variation regardless of drug infusion [44]. Removing the gallbladder data (Fig. 3e) results in complete separation of control and rifampicin subjects into two components (Fig. 3d), meaning there are no significant correlations between the two groups (*r>0.65, p<0.05*). The removal of gallbladder data makes this an informed method, as successful differentiation relies on curating the dataset.

### Uninformed intrasubject Δ*PCC* networks detect liver as region of interest

**Fig. 4:**
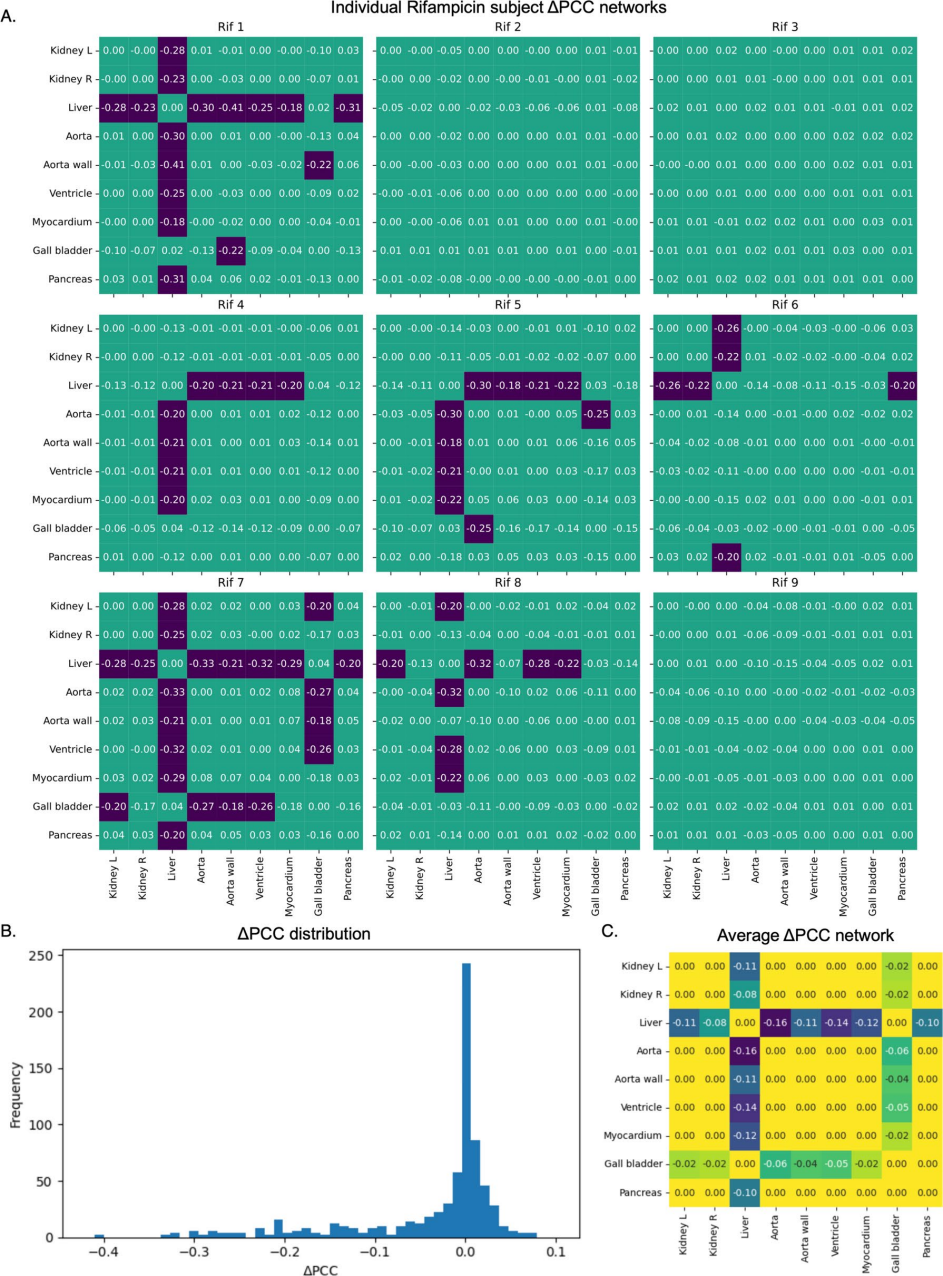
Intrasubject Δ*PCC* networks **a** Individual Δ*PCC* networks for each rifampicin subject **b** A histogram of all Δ*PCC* values across all regions in every rifampicin subject. Most values centre around 0, as most regions do not have a significant change in uptake before and after rifampicin infusion **c** The average of the individual Δ*PCC* networks, after thresholding for significance (|*ΔPCC*|*>0.17*). Only the liver and gallbladder show significant change between trial groups, with the liver displaying larger, more significant change

Unlike the first two methods, the Δ*PCC* method analyses the correlations between individual regions rather than between subjects, making it an intrasubject method. Individual networks are produced for each rifampicin subject (Fig. 4a), and after a significance threshold is applied (*|ΔPCC|>0.17, p<0.05*), an average network is created for the whole group (Fig. 4c). The individual networks, and the histogram displaying all Δ*PCC* values for all regions and subjects (Fig. 4b) show that most values centre around 0, such that the Pearson did not change significantly when the rifampicin subject was added to the controls. Values in purple in Fig. 4a are beyond the significance threshold. The only correlations that change significantly after adding in a rifampicin subject are the correlations of the liver and gallbladder to other regions. Not all subjects have significant Δ*PCC* values for the liver and gallbladder, but no subjects have significant Δ*PCC* values beyond these regions. In the average network (Fig. 4c), only significant values are maintained. The network displays significant variation in the gallbladder correlations with all other regions after rifampicin infusion, but the liver has more strongly significant Δ*PCC* values. This method is uninformed, as all data is included, and no curation is required to extract useful information from the networks.

## Discussion

Network analysis with [^11^C]glyburide WBPET in humans before and after infusion of rifampicin has been performed here to expand upon the use cases and outputs of different methods for network analysis with single-organ or whole-body research. This study can inform future research, guiding the application of network analysis with WB and TBPET data. Each of our methods were successful at stratifying the data, to varying degrees. All three methods have different potential research applications, useful for different PET datasets, level of knowledge of the clinical data, and the output target.

In the first method, single-organ or single-tissue *d-*networks are created with dynamic data, allowing for direct comparison of radiotracer kinetics in one region. This can be used to compare subjects’ responses to stimuli over a certain time course – such as rifampicin, in this study. Because the *d-*networks involve looking at data only from a predefined target tissue, it is an informed method. Here, we look at the liver specifically, because it is known to be the main region of [^11^C]glyburide uptake in the body [9, 18]. When we look at only the liver with this method, we see that network analysis separates the control and rifampicin groups into two separate halves of the network, but there are still some (above threshold) correlations between the two groups (Fig. 2a).

When we apply the method to a region that is not a direct target of [^11^C]glyburide and OATP expression, such as the blood pool, we see that there is no meaningful separation in the network (Fig. 2c) [9]. While there are still many significant correlations between individual’s data, the treatment group they belong to has no relevance to the *d-*network organisation. This would suggest that, regardless of rifampicin infusion, subjects have similar [^11^C]glyburide distribution in the blood over the course of the scan. That is, rifampicin infusion does not have a significant impact on blood exposure. This is particularly interesting in the context of previous research, which found a significant change in the areas under the TACs (AUC) in the blood, suggesting that the decrease in liver exposure led to increased blood exposure [9]. Because the *d-*network, which relies on *PCC*, compares the time-dependent changes in data rather than a time-average, it produces a different result than the AUC analysis. In effect, *PCC* measures the strength and direction of the linear correlation, but not the slope. This shows that, although the blood exposure was increased, the actual underlying distribution mechanism likely did not change in the blood. In the liver, though, OATP inhibition with rifampicin affects the mechanism of [^11^C]glyburide uptake, which is then reflected in the network separation.

Furthermore, we see that the control half of the liver *d-*network is characterized by denser, more interconnected nodes whereas the rifampicin side has fewer, slightly weaker connections. This could imply that not only does rifampicin cause a significant change in liver [^11^C]glyburide uptake from baseline, but that it also leads to less similar behaviour between rifampicin subjects. Assuming complete OATP inhibition and limited tissue diffusion of [^11^C]glyburide, this may reflect differences in the liver vasculature between subjects. Otherwise, this may reflect different levels of OATP inhibition across subjects following the same dose of rifampicin. In this way, the *d-*networks have now uncovered a potential area for further study. This method not only successfully distinguished the treatment groups into two halves of a network, but the topology – or arrangement of nodes – has also informed our perception of the similarities and differences in tracer uptake behaviour across subjects within each group.

In the second network analysis method, *s-*networks are created with static data. This method benefits from using more regions than the *d-*network method; however, it is also an informed method because the specific regions included must be chosen carefully based on physiology and prior knowledge of the tracer kinetics. Here, for example, the network shows no distinction between the two groups when all data is included (Fig. 3a) but shows complete separation into two components when the gallbladder data is removed (Figure 3C). Removing this data is reasonable, as [^11^C]glyburide is predominantly eliminated as metabolites and radioactivity in the gallbladder may depend on the activity of metabolic enzymes [15]. Furthermore, gallbladder behaviour is known to vary not only person to person, but also hour to hour within one subject. This is due to the nature of the gallbladder, which stores bile produced by the liver until it is needed in the duodenum [44]. Digestion was not a controlled factor in this protocol, and thus gallbladder emptying is expected to vary between all subjects. This can be seen by looking at the reference matrix *PCC*_*n*_ in Fig. 1, which is comprised of data only from the control group. The gallbladder typically has lower Pearson correlations than the other regions, as it is more variable between subjects. As the gallbladder is likely to be associated with eliminating glyburide from the body regardless of OATP expression, and its behaviour is expected to vary between all subjects regardless of treatment group, removing the gallbladder data from analysis is reasonable.

When the gallbladder data is removed, these informed *s-*networks separate the control and rifampicin groups to an even higher degree than the *d-*networks. Although this method cannot compare dynamic information, it does compare “fingerprints” of subjects – a snapshot of data from across the whole-body image altogether. This method harnesses the whole-body imaging approach and allows for comparisons to be made at the subject-level. These *s*-networks could be used to compare subjects from different known groups, such as the control and rifampicin groups, to detect key underlying physiological differences. Additionally, this method detects subgroups well and may be useful for discovering unknown subgroups within a larger cohort. This could provide potential diagnostic value by assessing where a subject belongs in the network. One example of this already exists, where the method was applied to patients with stage IIIB non-small cell lung carcinoma treated with chemotherapy, and the network organised the patients into three groups found to correlate with survival rate [32].

The third and final method, which creates Δ*PCC* networks, does not have the same limitations as the first two methods. It is uninformed, because it has no reliance on which regions are included or their order. The variability of the gallbladder is handled intrinsically. The Δ*PCC* network utilises static data, first measuring the correlations between every region and each other region within the control group and then assessing how those correlations change when a rifampicin subject is added in. Whereas significance in the *d-* and *s-*networks are directly determined by PCC significance, it is more nuanced for Δ*PCC*. The significance of Δ*PCC* depends not only on the number of subjects, *n*, but also on the distribution of *PCC*_*n*_. When *PCC*_*n*_*=0* with *n=13* control subjects, |Δ*PCC|>0.18* is significant at *p<0.05*. For increasing values of *PCC*_*n*,_ this threshold decreases. Here, we use |Δ*PCC|>0.18* as our significance level, and it is apparent from Fig. 4b that most values fall below threshold*.* The method for determining Δ*PCC* significance is expanded upon further in Supplementary Note S1 (Fig. S4-S7, Table S1), building off an existing method [45, 46].

While Δ*PCC* networks cannot be used to compare individual subjects, it is extremely useful for group-level research and detecting the region-level differences between trial groups. Here, the Δ*PCC* network successfully detects both the liver and gallbladder as important regions that vary between groups while still highlighting the liver as having more significant variation (Fig. 4c). In fact, this method manages to detect differences at the group-level even when individual subjects within the group may not have significant changes, such as rifampicin subjects 2, 3, and 9 in Fig. 4a. Interestingly, even with only 9 rifampicin subjects, the lack of significant change from these 3 subjects does not reduce the significance of the final results – showing that this method can be informative for group-level analysis even with smaller cohorts.

In this way, *s-*networks and Δ*PCC* networks could be very useful together, with Δ*PCC* networks highlighting physiological phenomenon which could inform the data included in creating *s-*networks. Furthermore, the subgroups detected by *s-*networks could be better understood with Δ*PCC* networks, which may be better suited to highlighting systemic physiological differences between groups. Finally, *d-*networks are then helpful, when dynamic data is available, for analysing these key physiological phenomena at a detailed, single-tissue level. These results are summarized in Table 1.

**Table 1:** Summary of the three network analysis methods

Method	*d-*networks	*s-*networks	*ΔPCC* networks
Data type	Dynamic	Static	Static
Prior knowledge	Networks specific to a region	Gall bladder data removed from consideration	Data separated by cohort, all regions included
Differentiation success	Differentiates in one region (liver), not others; Incomplete differentiation, some intergroup correlation	100% differentiation into two components	Sensitive to changes in both the liver (stronger) and gall bladder (weaker)
Research use	Dynamic data; Group-level analysis; Kinetic studies	Large dataset analysis; Group-level analysis; Uncovering group characteristics	Individual-level analysis; Uncovering what makes a subject (or subjects) different from the group

Each of the three network analysis methods presented here – *d-*networks, *s-*networks, and *ΔPCC* networks – rely on different levels of prior knowledge and may be useful in different research scenarios.

There are some limitations of the experiment which may affect the network analysis results; namely, that the experimental setup relies on a strong change between conditions and includes a small sample size of 22 scans. The strong change between the control and rifampicin groups makes this dataset a nice model for technique development, but it does not display how networks may or may not be sensitive to subtler differences in datasets. Studies have been conducted employing network analysis with less apparent differences in the data caused by the experimental paradigm [32, 34, 36]. These studies show the sensitivity of network analysis and how it compares to SUV analysis. Furthermore, previous work has been done to explore the robustness of network analysis methodology employed here, along with the statistical significance of network and correlation analysis with reduced datasets [34, 45].

Regardless, for further generalizability of the results, it would be ideal to conduct the analysis again with a larger cohort size. All networks shown here are based on statistically significant correlations for the sample size, but larger cohorts increase the significance of biological inferences. Increased cohort size would also allow for comparisons across sex and age, but this dataset did not show any trends within these groups at present. This may change with cohort size, as previously published work has suggested sex, but not age, has an impact on baseline OATP expression [18]. Furthermore, with increased cohort size, statistical characterisations of networks such as centrality, cliques, and more become more powerful. This can provide a more significant analysis of communities in a network. For example, with the liver *d-*network, these characterisations could allow for a more in-depth analysis of the difference in densities qualitatively observed between the control (denser) and rifampicin (less dense) groups.

Finally, although Pearson correlation has been used extensively in metabolic connectivity work to date, it relies on the assumption that the datasets being compared have a linear relation. We explore the validity of this assumption in supplementary material by comparing a linear fit versus a non-linear, monotonic fit of the data (Supplementary Table S2). This returns the linear fit as the more appropriate choice. We also present a liver-specific *d-*network made with Spearman correlation in supplementary material for comparison, which gives similar differentiation to the Pearson network but with fewer edges (Supplementary Fig. S8).

## Conclusion

The three methods of network analysis described here provide statistically significant ways of analysing WBPET data in humans, even with a traditionally small imaging sample of 22 scans. These methods not only display different results from SUV analysis, which has been discussed more in depth in other research [32, 34, 36], but also each provides a unique approach with different results from each other. The *d-*networks use dynamic data, are informed, and compare organs or tissues from different subjects. The *s-*networks use static data, are informed, and compare subjects. The Δ*PCC* networks use static data, are uninformed, and compare both at the organ-level and group-level. Each of these methods aligned with expected results when applied to [^11^C]glyburide WBPET pre- and post-infusion of rifampicin, while also revealing patterns in the data not necessarily seen through other methods of analysis. Further application of *d-*networks, *s-*networks, and Δ*PCC* networks with whole-body and total-body PET data could reveal novel information about physiology at any level, including tissue, systemic, and group analysis.

## Supplementary Information

Additional file 1.

## Data Availability

The datasets analysed during the current study are available from the corresponding author on reasonable request.

## Abbreviations

AUC: Areas under the TAC
CSV: Comma separated values
MRI & MR: Magnetic resonance imaging
OATP: Organic anion-transporting polypeptide
PCC: Pearson correlation coefficient
PET: Positron emission tomography
SUV: Standard uptake value
TAC: Time-activity curve
TBPET: Total-body PET
VOI: Volume of interest
WBPET: Whole-body PET
WB-4D PET: Whole-body dynamic PET

## References

[1] Rahmim A, Lodge MA, Karakatsanis NA, Panin VY, Zhou Y, McMillan A, et al. Dynamic whole-body PET imaging: principles, potentials and applications. Eur J Nucl Med Mol Imaging. 2019;46(2):501–18. 30269154 10.1007/s00259-018-4153-6

[2] Sundar LKS, Hacker M, Beyer T. Whole-body PET imaging: a catalyst for whole-person research? J Nucl Med. 2023;64(2):197–9. 10.2967/jnumed.122.264555. 36460342 10.2967/jnumed.122.264555 PMC9902855

[3] Burt T, Young G, Lee W, Kusuhara H, Langer O, Rowland M, et al. Phase 0/microdosing approaches: time for mainstream application in drug development? Nat Rev Drug Discov. 2020;19(11):801–18. 10.1038/s41573-020-0080-x. 32901140 10.1038/s41573-020-0080-x

[4] Tournier N, Stieger B, Langer O. Imaging techniques to study drug transporter function in vivo. Pharmacol Ther. 2018;189:104–22. 29684469 10.1016/j.pharmthera.2018.04.006

[5] McFeely SJ, Wu L, Ritchie TK, Unadkat J. Organic anion transporting polypeptide 2B1 – more than a glass-full of drug interactions. Pharmacol Ther. 2019;196(1):204–15. 30557631 10.1016/j.pharmthera.2018.12.009

[6] Nigam SK. What do drug transporters really do? Nat Rev Drug Discov. 2015;14(1):29–44. 25475361 10.1038/nrd4461 PMC4750486

[7] International Transporter Consortium, Giacomini KM, Huang SM, Tweedie DJ, Benet LZ, Brouwer KLR, et al. Membrane transporters in drug development. Nat Rev Drug Discov. 2010;9(3):215–36. 20190787 10.1038/nrd3028 PMC3326076

[8] Kusuhara H, Sugiyama Y. Role of transporters in the tissue-selective distribution and elimination of drugs: transporters in the liver, small intestine, brain and kidney. J Control Release. 2002;78(17):43–54. 11772448 10.1016/s0168-3659(01)00480-1

[9] Marie S, Breuil L, Chalampalakis Z, Becquemont L, Verstuyft C, Lecoq AL, et al. [^11^C]glyburide PET imaging for quantitative determination of the importance of organic anion-transporting polypeptide transporter function in the human liver and whole-body. Biomed Pharmacother. 2022. 10.1016/j.biopha.2022.113994. 36411655 10.1016/j.biopha.2022.113994

[10] Kovacsics D, Patik I, Özvegy-Laczka C. The role of organic anion transporting polypeptides in drug absorption, distribution, excretion and drug-drug interactions. Expert Opin Drug Metab Toxicol. 2017;13(4):409–24. 27783531 10.1080/17425255.2017.1253679

[11] Kim M, Deacon P, Tirona RG, Kim RB, Pin CL, Meyer zu Schwabedissen HE, et al. Characterization of OATP1B3 and OATP2B1 transporter expression in the islet of the adult human pancreas. Histochem Cell Biol. 2017;148(4):345–57. 28493059 10.1007/s00418-017-1580-6

[12] Roth M, Obaidat A, Hagenbuch B. OATPs, OATs and OCTs: the organic anion and cation transporters of the SLCO and SLC22A gene superfamilies. Br J Pharmacol. 2012;165(5):1260–87. 10.1111/j.1476-5381.2011.01724.x. 22013971 10.1111/j.1476-5381.2011.01724.x PMC3372714

[13] Zheng H, Huang Y, Frassetto L, Benet L. Elucidating rifampin’s inducing and inhibiting effects on glyburide pharmacokinetics and blood glucose in healthy volunteers: unmasking the differential effects of enzyme induction and transporter inhibition for a drug and its primary metabolite. Clin Pharmacol Ther. 2009;85(1):78–85. 18843263 10.1038/clpt.2008.186 PMC3582657

[14] Vavricka SR, Van Montfoort J, Ha HR, Meier PJ, Fattinger K. Interactions of rifamycin SV and rifampicin with organic anion uptake systems of human liver. Hepatology. 2002;36(1):164–72. 12085361 10.1053/jhep.2002.34133

[15] Li R, an Bi Y, Vildhede A, Scialis RJ, Mathialagan S, Yang X, et al. Transporter-Mediated Disposition, Clinical Pharmacokinetics and Cholestatic Potential of Glyburide and Its Primary Active Metabolites. Drug Metab Dispos. 2017;45(7):737–47. 28438781 10.1124/dmd.116.074815

[16] Koenen A, Köck K, Keiser M, Siegmund W, Kroemer HK, Grube M. Steroid hormones specifically modify the activity of organic anion transporting polypeptides. Eur J Pharm Sci. 2012;47(4):774–80. 22982504 10.1016/j.ejps.2012.08.017

[17] Satoh H, Yamashita F, Tsujimoto M, Murakami H, Koyabu N, Ohtani H, et al. Citrus juices inhibit the function of human organic anion-transporting polypeptide OATP-B. Drug Metab Dispos. 2005;33:518–23. 15640378 10.1124/dmd.104.002337

[18] Marie S, Lecoq A-L, Breuil L, Caillé F, Lebon V, Comtat C, et al. Imaging the impact of sex and age on OATP function in humans: consequences for whole-body pharmacokinetics and liver exposure. Acta Pharm Sin B. 2025;15(5):2736–45. 10.1016/j.apsb.2025.03.030. 40487663 10.1016/j.apsb.2025.03.030 PMC12144993

[19] Bullmore ET, Sporns O. Complex brain networks: graph theoretical analysis of structural and functional systems. Nat Rev Neurosci. 2009;10(3):186–98. 19190637 10.1038/nrn2575

[20] Yakushev I, Drzezga A, Habeck C. Metabolic connectivity: methods and applications. Curr Opin Neurol. 2017;30(6):677. 28914733 10.1097/WCO.0000000000000494

[21] Hahn A, Lanzenberger R, Kasper S. Making sense of connectivity. Int J Neuropsychopharmacol. 2019;22(3):194–207. 30544240 10.1093/ijnp/pyy100 PMC6403091

[22] Veronese M, Moro L, Arcolin M, Dipasquale O, Rizzo G, Expert P, et al. Covariance statistics and network analysis of brain PET imaging studies. Sci Rep. 2019;9(1):2496. 30792460 10.1038/s41598-019-39005-8 PMC6385265

[23] Shim HK, Lee HJ, Kim SE, Lee BI, Park S, Park KM. Alterations in the metabolic networks of temporal lobe epilepsy patients: a graph theoretical analysis using FDG-PET. NeuroImage: Clinical. 2020;27:102349. 32702626 10.1016/j.nicl.2020.102349 PMC7374556

[24] Sala A, Perani D. Brain molecular connectivity in neurodegenerative diseases: recent advances and new perspectives using positron emission tomography. Front Neurosci. 2019;13:617. 31258466 10.3389/fnins.2019.00617 PMC6587303

[25] Tuan PM, Adel M, Trung NL, Horowitz T, Parlak IB, Guedj E. FDG-PET-based brain network analysis: a brief review of metabolic connectivity. EJNMMI Rep. 2025;9(1):4. 39828812 10.1186/s41824-024-00232-6 PMC11743410

[26] Sanchez-Catasus CA, Müller MLTM, De Deyn PP, Dierckx RAJO, Bohnen NI, Melie-Garcia L. Use of Nuclear Medicine Molecular Neuroimaging to Model Brain Molecular Connectivity. In: PET and SPECT in Neurology. Springer, Cham; 2021. p. 181–207.

[27] Sánchez-Catasús CA, Sanabria-Diaz G, Willemsen A, Martinez-Montes E, Samper-Noa J, Aguila-Ruiz A, et al. Subtle alterations in cerebrovascular reactivity in mild cognitive impairment detected by graph theoretical analysis and not by the standard approach. Neuroimage Clin. 2017;15:160. 10.1016/j.nicl.2017.04.019 PMC5429238 28529871

[28] Bashan A, Bartsch RP, Kantelhardt JW, Havlin S, Ivanov PC. Network physiology reveals relations between network topology and physiological function. Nat Commun. 2012;3(1):702. 22426223 10.1038/ncomms1705 PMC3518900

[29] Reed MB, Ponce de León M, Vraka C, Rausch I, Godbersen GM, Popper V, et al. Whole-body metabolic connectivity framework with functional PET. Neuroimage. 2023;271:120030. 36925087 10.1016/j.neuroimage.2023.120030

[30] Ruan J, Wu Y, Wang H, Huang Z, Liu Z, Yang X, et al. Graph theory analysis of a human body metabolic network: a systematic and organ-specific study. Med Phys. 2025;52(4):2340–55. 10.1002/mp.17568. 39680791 10.1002/mp.17568

[31] Sun T, Wang Z, Wu Y, Gu F, Li X, Bai Y, et al. Identifying the individual metabolic abnormities from a systemic perspective using whole-body PET imaging. Eur J Nucl Med Mol Imaging. 2022;49(8):2994–3004. 35567627 10.1007/s00259-022-05832-7 PMC9106794

[32] Ronghe R, Crespo Gonzalez T, Wimberley C, Suchacki K, Tavares AAS. Innate [18F]fluorodeoxyglucose PET bone networks of lung cancer patients predict survival. Eur J Nucl Med Mol Imaging. 2025;52(13):4952–62. 10.1007/s00259-025-07388-8. 40471319 10.1007/s00259-025-07388-8 PMC12589343

[33] Sun L, Wu Y, Yang J, Liang J, Li P, Yu X, et al. The brain-white adipose tissue axis may play a crucial role in diabetes mellitus: a metabolic network analysis using total-body PET/CT imaging. Eur J Nucl Med Mol Imaging. 2025;52(13):5150–64. 10.1007/s00259-025-07337-5. 40392299 10.1007/s00259-025-07337-5

[34] Hellman AF, Clegg PS, Farquharson C, Millán JL, Alcaide-Corral CJ, Suchacki KJ, et al. Distinct bone metabolic networks identified in Phospho1−/− mice vs. wild type mice using [18F]FDG total-body PET. Front Med. 2025. 10.3389/fmed.2025.1597844. 10.3389/fmed.2025.1597844 PMC12133898 40470038

[35] Yu J, Spielvogel C, Haberl D, Jiang Z, Özer Ö, Pusitz S, et al. Systemic metabolic and volumetric assessment via whole-body [18F]FDG-PET/CT: pancreas size predicts cachexia in head and neck squamous cell carcinoma. Cancer. 2024;16(19):3352. 10.3390/cancers16193352 PMC11475137 39409971

[36] Suchacki KJ, Alcaide-Corral CJ, Nimale S, Macaskill MG, Stimson RH, Farquharson C, et al. A systems-level analysis of total-body PET data reveals complex skeletal metabolism networks in vivo. Front Med. 2021. 10.3389/fmed.2021.740615. 10.3389/fmed.2021.740615 PMC8488174 34616758

[37] Dias AH, Hansen AK, Munk OL, Gormsen LC. Normal values for 18F-FDG uptake in organs and tissues measured by dynamic whole body multiparametric FDG PET in 126 patients. EJNMMI Res. 2022;12:15. 35254514 10.1186/s13550-022-00884-0 PMC8901901

[38] Reed MB, Cocchi L, Sander CY, Chen J, Matheson GJ, Fisher P, et al. Connecting the dots: approaching a standardized nomenclature for molecular connectivity in positron emission tomography. Eur J Nucl Med Mol Imaging. 2025. 10.1007/s00259-025-07357-1. 40455254 10.1007/s00259-025-07357-1 PMC12660352

[39] Tuan PM, Horowitz T, Adel M, Wojak J, Trung NL, Guedj E. Comparative evaluation of graph construction methods for individual brain metabolic network from FDG-PET images: an ADNI study in healthy subjects. Eur J Nucl Med Mol Imaging. 2025 Aug 7. 10.1007/s00259-025-07462-1 40773019

[40] Freeman TC, Horsewell S, Patir A, Harling-Lee J, Regan T, Shih BB, et al. Graphia: a platform for the graph-based visualisation and analysis of high dimensional data. PLoS Comput Biol. 2022. 10.1371/journal.pcbi.1010310. 35877685 10.1371/journal.pcbi.1010310 PMC9352203

[41] Ch Kolaczyk ED. 7 Network Topology Inference. In: Kolaczyk ED, editor. Stat Anal Netw Data Methods Models. New York, NY: Springer; 2009. p. 1–48.

[42] Altman DG. Ch. 11.7 Correlation - Mathematics and Worked Examples. In: Altman DG, editor. Pract Stat Med Res. Milton, UK: CRC Press LLC; 1990. p. 293–4.

[43] Altman NS. An introduction to kernel and nearest-neighbor nonparametric regression. Am Stat. 1992;46(3):175–85. 10.1080/00031305.1992.10475879.

[44] Hall JE, Hall ME. Ch. 65. In: Hall JE, Hall ME, editors. Guyton and Hall Textbook of Medical Physiology. 14th ed. Elsevier; 2021.

[45] Liu X, Wang Y, Ji H, Aihara K, Chen L. Personalized characterization of diseases using sample-specific networks. Nucleic Acids Res. 2016;44(22):e164. 27596597 10.1093/nar/gkw772 PMC5159538

[46] Altman DG. Practical statistics for medical research. Milton, UK: CRC Press LLC; 1990.

