# Supplementary material for: Comparing three methodologies for network analysis of human [11C]glyburide whole-body PET data: d-networks, s-networks, and ΔPCC networks
Source: EJNMMI Res. 2025 Dec 31;16:24. doi: 10.1186/s13550-025-01348-x (PMC12886587; doi:10.1186/s13550-025-01348-x)
Supplement: Supplementary file 1 — Additional file 1. [file 13550_2025_1348_MOESM1_ESM.docx]

**Supplementary Material:** Comparing three methodologies for network analysis of human [^11^C]glyburide whole-body PET data: *d-*networks, *s-*networks, and *ΔPCC* networks

**Authors:** Abigail F. Hellman^1*^, Paul S. Clegg^1^, Solène Marie^2^, Nicolas Tournier^2^, Adriana A. S. Tavares^3,4^

**Affiliations:** ^1^School of Physics and Astronomy, University of Edinburgh, Edinburgh, United Kingdom; ^2^Université Paris-Saclay, CEA, Inserm, CNRS, BioMaps, Service Hospitalier Frédéric Joliot, Orsay, France; ^3^University/British Heart Foundation (BHF) Centre for Cardiovascular Science, The Queen’s Medical Research Institute, University of Edinburgh, Edinburgh, United Kingdom; ^4^Edinburgh Imaging, University of Edinburgh, Edinburgh, United Kingdom

[*a.f.hellman@ed.ac.uk](mailto:a.f.hellman@ed.ac.uk)

**Data styles and method diagrams**

**Fig. S1** Method for deriving *d-*networks, which compare between subjects using dynamic data at a single-region level


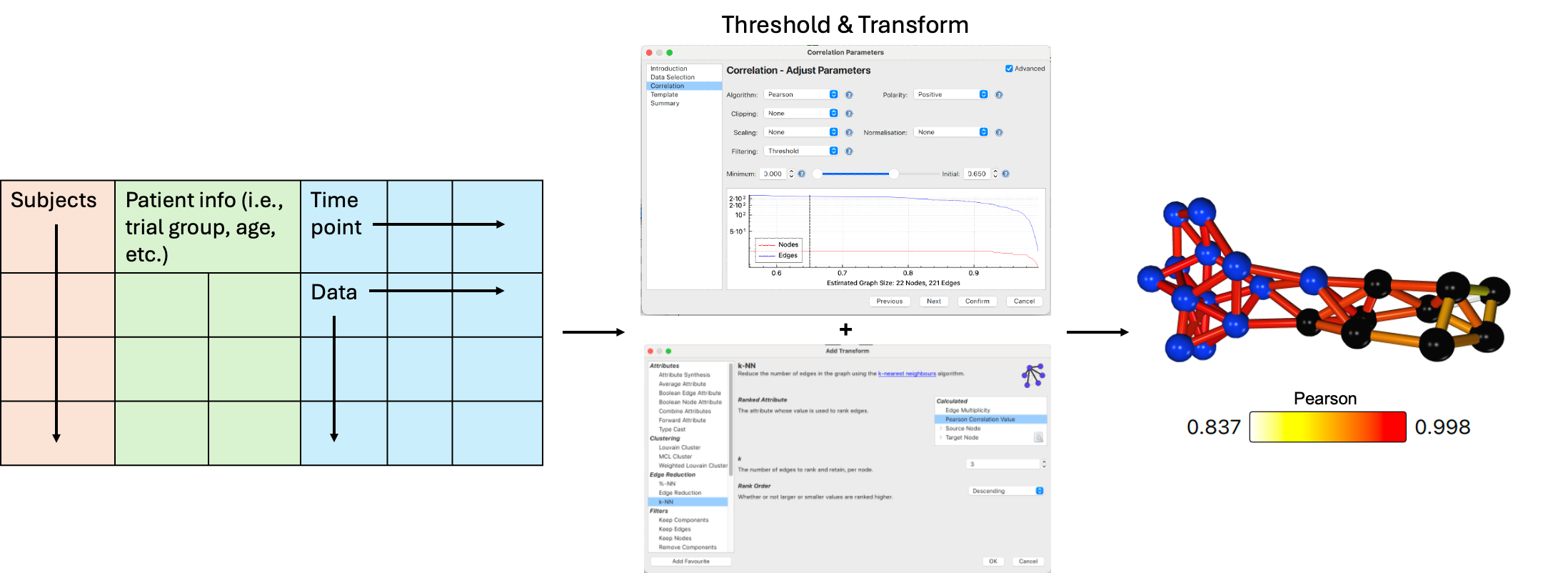


**Fig. S2** Method for deriving *s-*networks, which compare between subjects using static data and included data from multiple regions


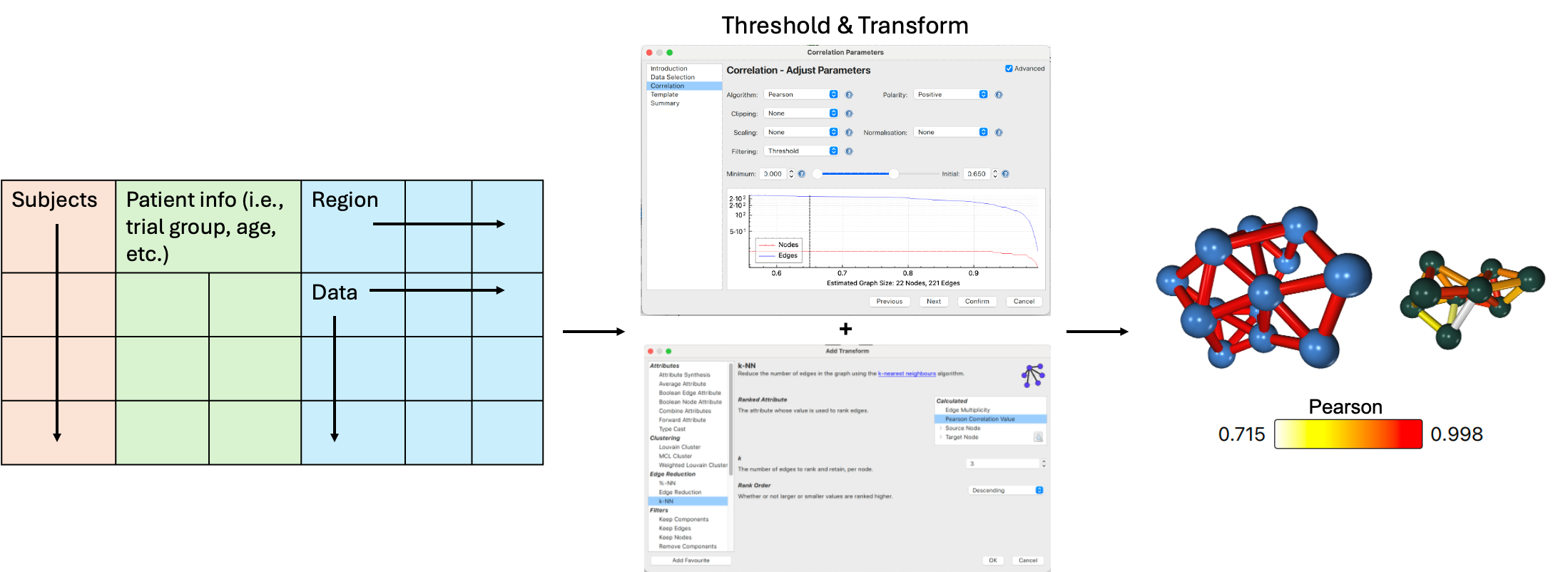


**Intersubject d-networks for separate regions of interest**

**Fig. S3** Intersubject *d-*networks where each node represents an SUV TAC from a single subject for the respective organ (green is control, orange is rifampicin). These networks compare between subjects at a single-region level

**Note S1: Determination of significance thresholds**

Problem statement:

Networks analysis can be carried out by exploring changes to the correlation patterns for the control subjects compared to the correlation patterns for the control subjects with one of the treatment subjects included [1]. The change in the Pearson correlation coefficient is usually evaluated:

$$\Delta{PCC}_{n}={PCC}_{n}-{PCC}_{n+1}$$

where $n$ is the number of control subjects, ${PCC}_{n}$is the Pearson correlation coefficient between activity values for two organs calculated for the control subjects and ${PCC}_{n+1}$ is the same quantity calculated for the control subjects with one of the treatment subjects additionally included.

We would like to test the null hypothesis that the observed value $\Delta{PCC}_{n}$is due to random chance where the underlying distributions of Pearson correlation coefficients are in fact identical. Unfortunately, the distribution of $\Delta{PCC}_{n}$is not described by any common distribution (e.g. normal or t distribution).

To solve this problem, we generate synthetic values for ${PCC}_{n}$and ${PCC}_{n+1}$ so that we can determine the distribution of $\Delta{PCC}_{n}$ values from identical underlying distributions. This will allow us to estimate a threshold value of $\Delta{PCC}_{n}$ for a significant change in the Pearson correlation coefficient.

The distribution of Pearson correlation coefficient values, $r$, about a characteristic ${PCC}_{n}$value is not normal. However, we can transform $r$ into the quantity

$$g=\frac{1}{2}\log_{e} \left( \frac{1+r}{1-r} \right)$$

which is normal [2] and has a standard error $s\approx1/\sqrt{n-3}$. Here $n$ remains the number of control subjects. Below we work with samples of $r$ values which exhibit these characteristics.

Simulation method:

In order to generate two arrays each containing $n$ values with the desired $r$ we draw the first array, $x$, from a zero-mean, unit-standard-deviation normal distribution. The second array, $y$, is then drawn from a normal distribution with the means $r\times x$ and the single standard deviation value $\sqrt{1- r^{2}}$ . The Pearson correlation coefficient between $x$ and $y$ is ${PCC}_{n}$.

To find ${PCC}_{n+1}$, we append one further value to each of the two arrays using these same distributions. I.e. apart from the lengths of the arrays, the characteristics of ${PCC}_{n}$and ${PCC}_{n+1}$are identical.

(1) We calculate 1 million $\Delta{PCC}_{n}$ values characteristic of a chosen ${PCC}_{n}$ value. We use the distribution to determine the $\Delta{PCC}_{n}$ values bounding the 2.5% and the 97.5% percentiles. These studies have been carried out for ${PCC}_{n}=0$ and also for a succession of non-zero values typically encountered in our networks (${PCC}_{n}=0.99, 0.94, 0.89, 0.84, 0.79, 0.74, 0.69, 0.64$). These simulations have been carried out for both $n=100$ and $n=13$. The latter is the size of the number of control subjects in our study.

(2) In order to study the behaviour as $n$ varies over a broad range, we have calculated 10 thousand $\Delta{PCC}_{n}$ values characteristic of a chosen ${PCC}_{n}$ value for $5\leq n\leq200.$ We plot the 2.5% and the 97.5% percentiles versus $n$.

Distributions:

At ${PCC}_{n}=0$ the distribution of $\Delta{PCC}_{n}$ is symmetric and sharply peaked about zero (Figure S2).


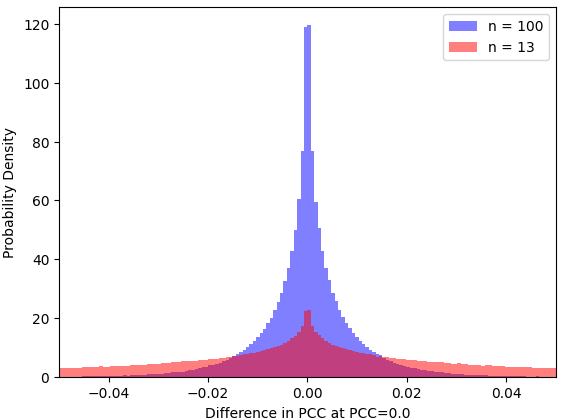


*Figure S4: The probability density distributions for* $\Delta{PCC}_{n}$ *for two* $n$ *values when* ${PCC}_{n}=0$*.*

As previously noted (Figure 2b in Ref. [1]), this distribution is far from normal. It is also evident that the spread of $\Delta{PCC}_{n}$ becomes much more pronounced as the size of the control group decreases.

At ${PCC}_{n}=0$ the variation in the significance threshold for $\Delta{PCC}_{n}$ across a wide range of $n$ values is shown in Figure S3. Because the $\Delta{PCC}_{n}$ distributions are symmetric we plot a single line (labelled observed) which is the average of the magnitudes of the 2.5% and the 97.5% percentiles.


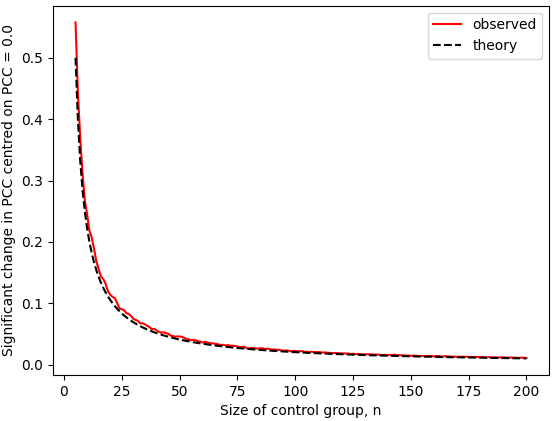


*Figure S5: Variation of the significance threshold for* $\Delta{PCC}_{n}$ *(i.e. the magnitude of the 2.5% percentile) as a function of,* $n$*, the number of subjects in the control group when* ${PCC}_{n}=0$*. The values observed in simulations are compared to those from a large* $n$*approximation (theory).*

As is expected, a smaller change is significant when the control group is very large. The dashed line labelled theory is determined from equation (2) in Ref. [1]. This is the large $n$ approximation:

$$z=\frac{\Delta{PCC}_{n}}{\frac{1- {{PCC}_{n}}^{2}}{n-1}}$$

At any values of ${PCC}_{n}$ and $n$ an estimate of a value $\Delta{PCC}_{n}$ that is significant at a p = 0.05 level can be found by setting $z = 1.96$. As would be expected for a large $n$ approximation, this describes the simulations nicely at large $n$ but increasingly poorly at small $n$ (Figure S3).

As an example of the other distributions, Figure S4 shows the spread of $\Delta{PCC}_{n}$ at ${PCC}_{n}=0.74$.


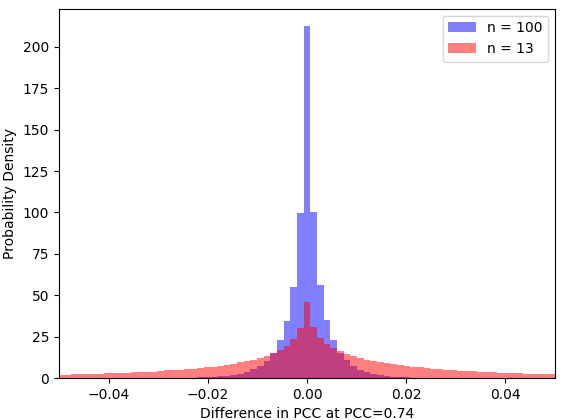


*Figure S6: The probability density distributions for* $\Delta{PCC}_{n}$ *for two* $n$ *values when* ${PCC}_{n}=0.74$*.*

At $n=100$, the tails of the distribution are noticeably less pronounced compared to ${PCC}_{n}=0$. For $n=13$ the distribution is slightly asymmetric. This effect is easier to see in the plot of the significance threshold for $\Delta{PCC}_{n}$ across a wide range of $n$ values shown in Figure S5.


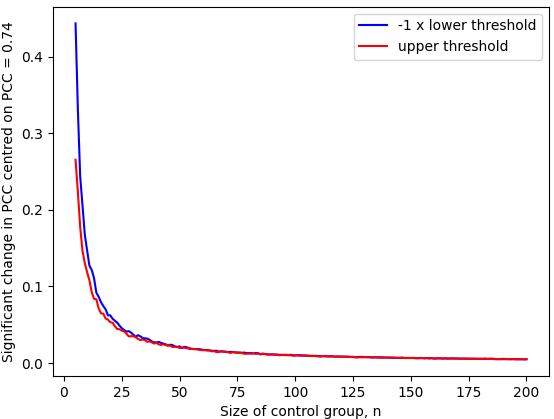


*Figure S7: Variation of the significance thresholds for* $\Delta{PCC}_{n}$ *(lower - the magnitude of the 2.5% percentile; upper – the 97.5% percentile) as a function of,* $n$*, the number of subjects in the control group when* ${PCC}_{n}=0.74$*. Asymmetry becomes increasingly obvious at low* $n$*.*

At very low $n$, the threshold for significance becomes strongly different for positive and negative values of $\Delta{PCC}_{n}$.

Significance values:

To guide network analysis, Table S1 presents the location of the 2.5% percentile of the simulated $\Delta{PCC}_{n}$ distributions for a range of ${PCC}_{n}$ values. (The 2.5% percentile is never smaller in magnitude than the 97.5% percentile and hence provides a conservative value for judging significant changes.) These are given for both $n=100$ and $n=13$. Additionally, the large $n$ approximation using $z = 1.96$ is also included for comparison. The magnitude of this approximation is always roughly correct but differs in detail by between 10% and 40% compared to the simulations at $n=13$.

| **PCC** | **2.5% (n=100)** | **z=1.96 (n=100)** | **2.5% (n=13)** | **z=1.96 (n=13)** |
| --- | --- | --- | --- | --- |
| 0.99 | -0.0005 | 0.0004 | -0.0053 | 0.0033 |
| 0.94 | -0.0027 | 0.0023 | -0.0297 | 0.0190 |
| 0.89 | -0.0048 | 0.0041 | -0.0514 | 0.0340 |
| 0.84 | -0.0067 | 0.0058 | -0.0710 | 0.0481 |
| 0.79 | -0.0085 | 0.0074 | -0.0879 | 0.0614 |
| 0.74 | -0.0103 | 0.0090 | -0.1038 | 0.0739 |
| 0.69 | -0.0118 | 0.0104 | -0.1164 | 0.0856 |
| 0.64 | -0.0134 | 0.0117 | -0.1289 | 0.0964 |
| 0.0 | -0.0219 | 0.0198 | -0.1816 | 0.1633 |

*Table S1: Suggested significance thresholds to use for* $\Delta{PCC}_{n}$ *when n=13 (column 4) for different* ${PCC}_{n}$ *values (column 1) compared to the magnitude of the large* $n$ *approximation (column 5) and estimates for* $n=100$ *(columns 2 and 3).*

The table above can be used to select an appropriate threshold for significance, below which values of $\Delta{PCC}_{n}$ are taken to be zero.

**Test of data linearity:**

**Table S2** Using the dynamic liver data and selecting one subject at random as the independent variable and performing linear and non-linear monotonic (one-phase decay and one-phase association) regressions with the other subjects as dependent variables shows that a linear fit is often more appropriate than a non-linear monotonic fit. The Comparisons of Fit were performed with an Extra Sum of Squares F Test (*p<0.05*). This would suggest that Pearson is an appropriate choice for correlation calculations with this dataset.

**Comparison use of Spearman rank correlation:**

**Fig. S8** The liver *d-*network (which compares between subjects at a single-region level) generated with Spearman rank correlation instead of Pearson correlation. The network still mostly separates based on group, except for one rifampicin subject on the control side of the network.

**References:**

[1] 40. Liu X, Wang Y, Ji H, Aihara K, Chen L. Personalized characterization of diseases using sample-specific networks. *Nucleic Acids Res*. 2016;44:e164.

[2] Altman DG. Practical Statistics for Medical Research. CRC Press LLC; 1990.
